# Alloy Design, Thermodynamics, and Electron Microscopy of Ternary Ti-Ag-Nb Alloy with Liquid Phase Separation

**DOI:** 10.3390/ma13225268

**Published:** 2020-11-21

**Authors:** Takeshi Nagase

**Affiliations:** 1 Research Center for Ultra-High Voltage Electron Microscopy, Osaka University,7-1, Mihogaoka, Ibaraki, Osaka 567-0047, Japan; t-nagase@uhvem.osaka-u.ac.jp; 2 Division of Materials and Manufacturing Science, Graduate School of Engineering, Osaka University, 2-1 Yamadaoka, Suita, Osaka 565-0871, Japan

**Keywords:** titanium alloys, solidification, microstructure, electron microscopy, thermodynamics

## Abstract

The Ti–Ag alloy system is an important constituent of dental casting materials and metallic biomaterials with antibacterial functions. The binary Ti–Ag alloy system is characterized by flat liquidus lines with metastable liquid miscibility gaps in the phase diagram. The ternary Ti–Ag-based alloys with liquid phase separation (LPS) were designed based on the mixing enthalpy parameters, thermodynamic calculations using FactSage and Scientific Group Thermodata Europe (SGTE) database, and the predicted ground state diagrams constructed by the Materials Project. The LPS behavior in the ternary Ti–Ag–Nb alloy was investigated using the solidification microstructure analysis in arc-melted ingots and rapidly solidified melt-spun ribbons via trans-scale observations, combined with optical microscopy (OM), scanning electron microscopy (SEM) including electron probe micro analysis (EPMA), transmission electron microscopy (TEM), and scanning transmission electron microscopy (STEM). The solidification microstructures depended on the solidification processing in ternary Ti–Ag–Nb alloys; macroscopic phase-separated structures were observed in the arc-melted ingots, whereas fine Ag globules embedded in the Ti-based matrix were observed in the melt-spun ribbons.

## 1. Introduction

Liquid phase separation (LPS) is commonly observed in various metallic materials, including Ti-based alloys. Past research focusing on LPS in Ti-based alloys can be summarized based on the following three categories. (1) Ti-rare earth-based alloys for the development of Ti-based alloys with fine globules, focusing on structural materials [1,2,3,4,5,6]. A number of binary Ti-rare earth alloy systems show a monolicic phase diagram with a liquid miscibility gap. (2) Ti–Mg-based immiscible alloys for lightweight materials [7,8]. Ti and Mg are immiscible despite Mg being in the liquid state. (3) Ti–Ag-based alloys focused on the occurrence of LPS [9,10] and the demand for dental materials and antibacterial materials. The Ti–Ag binary phase diagram is characterized by a flat liquidus and metastable liquid miscibility gap at temperatures below the liquidus temperature [9].

In Ti-rare earth-based alloys (1), many binary alloy systems of Ti–Y [11], Ti–La [3,12], Ti–Ce [13], Ti–Pr [14], Ti–Nd [15], Ti–Gd [16], Ti–Dy [17], and Ti–Yb [18] exhibit a monolicic phase diagram with a liquid miscibility gap in the phase diagram. Although the flat liquidus implies a metastable liquid miscibility gap in the Ti–Er alloy system, the occurrence of LPS in the Ti–Er alloy system with a flat liquidus line cannot be detected because of the formation of nonmetallic Er globules and Er_2_O_3_ oxide globules [6]. Focusing on the solidification microstructure of the Ti-rare earth-based immiscible alloys with LPS, fine metallic and/or oxide globules with dispersed microstructures have been observed [1,2,3,4,5,6]. The formation of fine globules, including the formation of metallic and/or oxide globules, was explained using the liquid miscibility gap and low solubility of rare-earth elements in Ti phase in the thermal equilibrium phase diagrams, and high oxidation tendency of rare-earth elements, among other causes. The dispersion of fine globules was reported to be effective for the strengthening of Ti-based alloys. However, a detailed discussion on the LPS has not been conducted regarding the Ti-rare earth-based alloys because of the significantly high oxidation tendency of the rare-earth elements and the difficulty in processing Ti-based alloy materials with the rare-earth elements. Little is considered about the alloy design of multi-component Ti-rare earth-based alloys with LPS.

In the Ti-Mg alloy system (2), it is known that Ti and Mg are immiscible despite Mg being in the liquid state [19]. The significantly large positive value of the mixing enthalpy (Δ*H*_i-j_) of the Ti_50_Mg_50_ alloy (16 kJ·mol^−1^) listed in the literature [20,21] demonstrates the immiscibility of the Ti and Mg liquids in Ti–Mg alloys. Currently, there is no evidence of LPS because of the difficulty in the experimental study and lack of thermodynamic data for the calculations. The microstructure of vapor-quenched Ti–Mg alloys has been reported, but the typical microstructure of LPS was not observed in Ti–Mg alloys [7,8].

The Ti–Ag alloy system (3) is characterized by a flat liquidus line in the binary phase diagram [22,23]. The occurrence of LPS during the rapid cooling of the thermal melt in binary Ti–Ag alloys due to the metastable liquid miscibility gap was detected using electron microscopy of the solidified structure [9]. This was also in line with the thermodynamic calculations [9]. Based on the practical application of Ti–Ag-based alloys, the microstructures of the Ti–Ag alloy ingots were investigated for their application as dental materials and further clarifying details about their biocompatibilities, corrosion resistances, mechanical properties, and machinabilities [24,25,26,27,28,29,30,31,32,33,34]. Ag is recognized as an important element that causes a decrease in the melting point of dental casting alloys [25,26]. Recently, Ti–Ag-based alloys were reported and investigated for their application as antibacterial materials [35,36,37,38,39,40]. The main route for the fabrication of Ti–Ag-based dental materials and metallic antibacterial materials used the casting process, with a dental casting machine and arc casting furnace. The microstructure of the ingots showed a conventional dendritic structure that included Ti–Ag-based intermetallic compounds without LPS. Wen et al. reported the microstructure of Ti–Nb–Ag (Ti–26Nb–5Ag) alloys fabricated using powder metallurgy for the development of new Ti-based metallic biomaterials, and pointed out that Ti alloys with microstructures dispersed with fine Ag phases can exhibit antibacterial properties [41]. Hence, an appropriate fabrication method to produce Ti–Ag-based alloys with fine Ag phases not only in binary alloy systems but also in multicomponent alloys is desired [41]. It has been reported that Ti-based alloys, including ternary alloys [42,43,44], show particular structural changes during mechanical alloying and cooling in nanoscale alloys. The successful fabrication of ternary and/or multicomponent Ti–Ag-based alloys containing fine Ag particles with LPS may offer a unique opportunity for the development of Ti–Ag-based dental and antibacterial materials. However, only a few studies on the behavior of LPS in Ti–Ag-based alloys have been reported to date [9,10]. In this study, the behavior of LPS in a ternary Ti–Ag-based alloy system of Ti–Ag–Nb was investigated from the viewpoint of the alloy design of ternary Ti–Ag-based alloys with LPS and the solidification microstructure characterization of the Ti–Ag–Nb ternary alloy with LPS.

## 2. Materials and Methods

Commercially available element chips of Ti (approximately 8 × 8 × 2 mm, Mitsuwa pure chemicals Co. Ltd., Osaka, Japan, purity = 3N), granules of Nb (2–5 mm, Mitsuwa pure chemicals Co. Ltd., Osaka, Japan, purity = 3N, and shots of Ag (2–6 mm, Nilaco Co. Ltd., Tokyo, Japan, purity = 3N) were used. The alloy compositions of Ti_66.7_Ag_33.3_ (corresponding to Ti_2_Ag) and Ti_53.4_Ag_33.3_Nb_13.3_ (corresponding to (Ti_0.8_Nb_0.2_)_2_Ag) were investigated. The arc-melted ingots were prepared from the mixture of Ti chips, Nb granules, and Ag shots of the pure elements. The cooling rate during the arc melting process was approximately 2 × 10^3^ K/s based on the secondary dendrite spacing in the Al–Cu alloy [9,45]. It should be noted here that the cooling rate during the arc-melting process was one order higher than that during the centrifugal metallic mold casting (approximately 200–600 K/s) [46] and three orders higher than that during the silica-based crucible cooling of the thermal melt (the order of 1 K/s) [47]. The rapidly quenched ribbons were produced from the master ingots using a single roller melt-spinning method. A fused quartz nozzle with a 14 mm diameter and 0.5 mm orifice was used, and the heating of the master ingot was conducted using the radio frequency. The roller surface velocity was approximately 42 m/s. The cooling rate of the single roller melt-spinning method was approximately 10^5^–10^6^ K/s [48,49]. The dependence of the cooling rate on the solidification microstructure was investigated by comparing the arc-melted ingots and melt-spun ribbons. One may consider applying various casting processes with different cooling rates for evaluating the cooling rate dependence. The molten state of Ti-based alloys shows high reactivity with the crucible materials and high oxidation tendency, resulting in limitations of the casting process for Ti-based alloys. In this study, only the arc-melting and melt-spinning processes were used. The solidification microstructures of the ingots and melt-spun ribbons were examined using X-ray diffraction (XRD) using Cu-Kα radiation and scanning electron microscopy (SEM)-backscattered electron (BSE) image observation, and electron probe microanalysis (EPMA)-wavelength dispersive X-ray spectrometry (WDS) analysis. Transmission electron microscopy (TEM) and scanning transmission electron microscopy (STEM) were performed using the Hitachi H-800 (Hitachi, Tokyo, Japan) and JEOL JEM-2100F systems (JEOL, Tokyo, Japan), respectively. The thin films for the TEM and STEM analyses were prepared using an ion thinning method using the Gatan (Gatan, Pleasanton, CA, USA) precision ion polishing system (PIPS, model 691). The LPS behavior in the ternary Ti–Ag–Nb alloy was investigated with the help of trans-scale observations, combined with various microscopy imaging techniques, including OM, SEM, EPMA, TEM, and STEM. The thermodynamic calculations were performed using FactSage ver7.3 [50] and the Scientific Group Thermodata Europe 2017 (SGTE2017) database [51]. In the SGTE2017 database, the binary phase diagrams of the Ti-Ag, Ti-Nb, and Ag-Nb alloy systems were accessed.

## 3. Alloy Design

**Figure 1:**
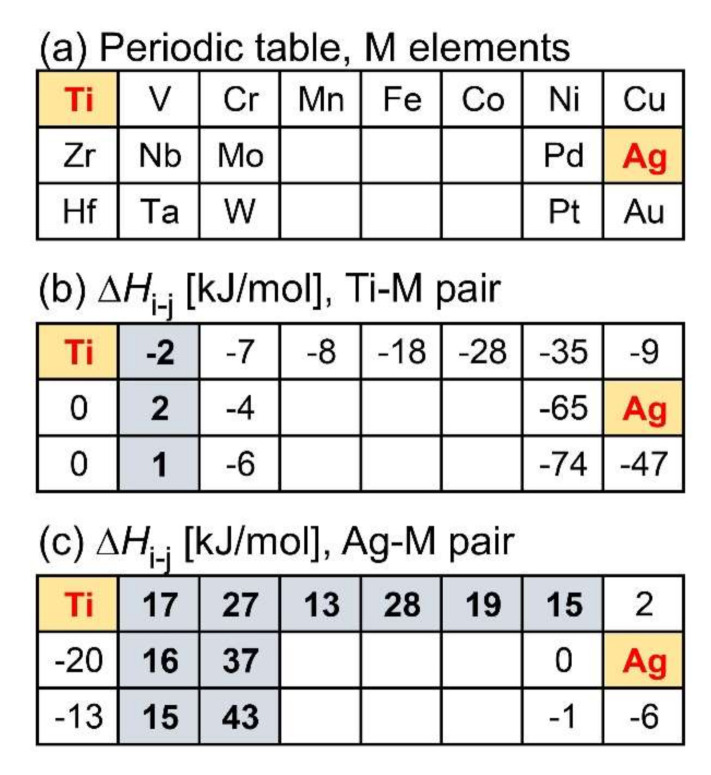
Periodic table and the value of Δ*H*_i-j_ in Ti–M and Ag–M atomic pairs for the alloy design of ternary Ti–Ag–M alloy with LPS. (**a**) Periodic table for considering M element in the ternary Ti–Ag–M alloy; (**b**) Δ*H*_i-j_ for the Ti–M atomic pair; (**c**) Δ*H*_i-j_ for the Ag–M atomic pair. The values of Δ*H*_i-j_ was obtained from the literature [20,21].

The values of Δ*H*_i-j_ shown in the references [20,21] were effective in predicting the LPS tendency in alloys. The alloy design technique using the matrix of Δ*H*_i-j_ among constituent elements shown in the literature [49] was effective in developing various multicomponent alloys with LPS, including quaternary metallic glasses (MGs) [52,53,54,55] and high-entropy alloys (HEAs) [56,57]. The approach of using the Δ*H*_i-j_ matrix for the design of ternary Ti–Ag–M alloys was adopted in the present study, and the results are shown in Figure 1. As shown in Figure 1a, ternary Ti–Ag–M alloy systems were considered for various elements (M). The elements in the blank spaces correspond to Tc, Re, Ru, Os, Rh, and Ir, and these elements are not discussed in the present study. This is because these are non-common elements for conventional Ti-based alloys. For the alloy design of ternary Ti–Ag–M alloys with LPS and the formation of an Ag-rich liquid, the following two conditions were favorable: (1) the low absolute value of Δ*H*_i-j_ in Ti–M, to suppress the formation of Ti–M-based intermetallic compounds and MGs, (2) large positive values of Δ*H*_i-j_ in Ag–M, for the occurrence of LPS and the formation of Ti–M-rich and Ag-rich liquids via LPS. The value of Δ*H*_i-j_ in Ti–Ag was −2 kJ/mol, which has already been discussed in detail in a previous paper [9], and, therefore, is not discussed in detail here. In the Ti−M atomic pair (Figure 1b), the pairs with low absolute values of Δ*H*_i-j_ under 2 kJ/mol (written in bold with a gray background) were for M = V, Nb, or Ta (where M is a group 5 element). In the Ag–M atomic pair (Figure 1c), the pairs with a large positive value of Δ*H*_i-j_ equal to and above 10 kJ/mol (written in bold with a gray background) were for M = V, Nb, Ta (group 5 elements); group 6 elements of Cr, Mo, W; 3D-transition metal elements of Cr, Mn, Fr, Co, and Ni. However, only the group 5 elements, V, Nb, and Ta, satisfied the simultaneous requirement conditions of a significantly small absolute value of Δ*H*_i-j_ in the Ti–M atomic pair and a large positive value of Δ*H*_i-j_ in the Ag–M atomic pair. From the viewpoint of the metallic biomaterials, V (vanadium) was found to be undesirable [58,59,60,61]. The melting temperature is an important factor for fabricating specimens via the solidification route. The melting temperature of Ta (3290 K) is much higher than that of Nb (2750 K). This indicates that it is more difficult to fabricate specimens in the ternary Ti–Ag–Ta alloy system than with the Ti–Ag–Nb alloy system. The ternary Ti–Ag–Nb alloy system, based on the above-described alloy design, is investigated in the present study.

**Figure 2:**
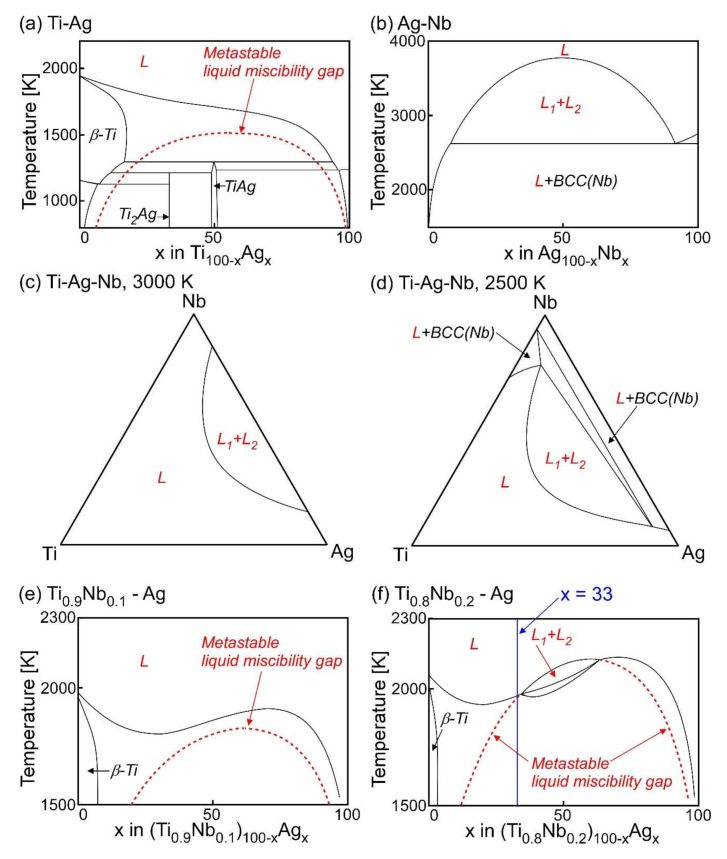
Thermodynamic calculation results of the Ti-Ag, Ag-Nb and Ti-Nb-Ag alloys. (**a**) Ti-Ag alloy with metastable liquid miscibility gap; (**b**) Ag-Nb alloy; (**c**) Liquidus projection in Ti-Nb-Ag alloy at 3000 K; (**d**) Liquidus projection in Ti-Nb-Ag alloy at 2500 K; (**e**) pseudo-binary Ti_0.9_Nb_0.1_-Ag alloy with metastable liquid miscibility gap; (**f**) pseudo-binary Ti_0.8_Nb_0.2_-Ag alloy with metastable liquid miscibility gap. The metastable liquid miscibility gap is represented by red broken lines.

The LPS tendency in the ternary Ti–Ag–Nb alloy system is discussed using thermodynamic calculations with the help of the FactSage and SGTE2017 databases, and the results are shown in Figure 2. In the Ti–Ag binary alloy system, a metastable liquid miscibility gap exists at temperatures below the flat liquidus [9]. Among the values of Δ*H*_i-j_ shown in Figure 1b, the atomic pair of Ag–Nb showed large positive values, which corresponded to the monotectic reaction in the calculated Ag–Nb phase diagram (Figure 2b). A binary Ag–Nb phase diagram covering all the composition ranges and wide temperature ranges has not been reported. However, some ternary phase diagrams that include binary Ag–Nb pairs have been reported [62,63]. No intermetallic compounds were observed in the binary Ag–Nb phase diagrams, and the calculated phase diagram shown in Figure 2b was consistent with the previous reports [62,63]. Figure 2c,d shows the region of the stable two-liquid phase region (L_1_ + L_2_) in the ternary Ti–Ag–Nb alloy system, indicating that the addition of Nb enhances the LPS tendency in the Ti–Ag and Ti–Ag–Nb alloy systems. The two liquid states shifted to the Ti-rich side in the Ti–Nb–Ag alloys with decreasing temperature, as shown in Figure 2c,d. Figure 2e shows the pseudo-binary Ti_0.9_Nb_0.1_–Ag alloy focusing on the liquidus and liquid miscibility gap. A metastable liquid miscibility gap was observed at temperatures below the liquidus in the Ti_0.9_Nb_0.1_–Ag alloy (Figure 2e), which was similar to the binary Ti–Ag alloy (Figure 2a). The pseudo-binary Ti_0.8_Nb_0.2_–Ag alloy focusing on the liquidus and liquid miscibility gap exhibits a stable L_1_ + L_2_ region, as shown in Figure 2f. Table 1 shows the results of the thermodynamic calculation of the composition of the separated liquids in the (Ti_0.8_Nb_0.2_)_2_Ag of (Ti_0.8_Nb_0.2_)_1−x_Ag_x_ (x = 0.33) (Ti_53.4_Ag_33.3_Nb_13.3_ at.%) alloy. The Ti-rich and Ag-rich liquids were formed via LPS in the Ti_53.4_Ag_33.3_Nb_13.3_ alloy. In the Ti-rich liquid (Table 1a), the concentration of Ag decreased with decreasing temperature. The concentrations of Ti and Nb decreased with decreasing temperature of the Ag-rich liquid (Table 1b). Nb showed a tendency to be enriched in the Ti-rich liquid rather than in the Ag-rich liquid. The formation of the Ti-Nb-rich and Ag-rich liquids via LPS was predicted using thermodynamic calculations of the Ti_53.4_Ag_33.3_Nb_13.3_ alloy.

**Figure 3:**
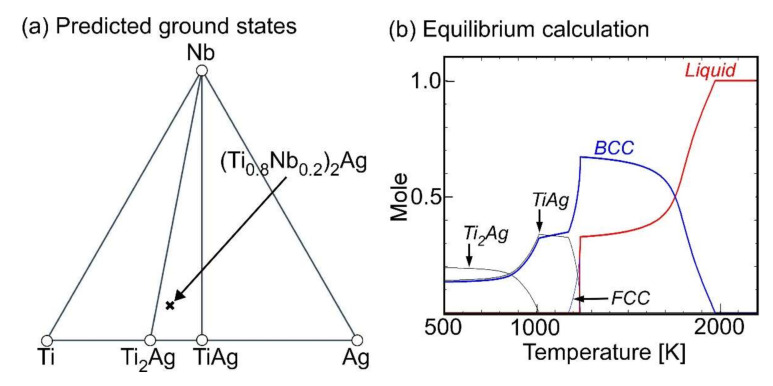
(**a**) Predicted ground states in ternary Ti–Ag–Nb alloy system constructed by the Materials Project, and (**b**) the equilibrium calculation in Ti_53.4_Ag_33.3_Nb_13.3_ alloy.

The possibility for the suppression of LPS by the formation of intermetallic compounds was investigated as per the predicted ground state diagram constructed using the Materials Project [64,65] and equilibrium calculations. The results are shown in Figure 3. The application of the predicted ground state diagram constructed using the Materials Project was found to be effective in designing multicomponent alloys with LPS, including LPS type quaternary MGs [52,53,54,55] and HEAs [56,57,66], even when the thermodynamic calculations were not available [63]. Figure 3a shows the predicted ground states in the ternary Ti–Ag–Nb alloy system. The predicted phase diagram (Figure 3a) showed the existence of TiAg [67] and TiAg_2_ [68] intermetallic compounds, which corresponded to the binary Ti–Ag phase diagram (Figure 2a). There are no intermetallic compounds in the binary Ti–Nb alloy system in Figure 3a, which corresponds to the existence of a BCC solid solution with a complete range of solubility and no intermetallic compounds in the binary Ti–Nb phase diagram [69,70,71]. Ternary Ti–Nb–Ag intermetallic compounds were not observed in the predicted phase diagram, which indicated that the formation of the ternary Ti–Nb–Ag intermetallic compounds in the Ti–Nb– Ag alloy system did not suppress LPS. It should be noted here that there are no ternary intermetallic compounds with congruent melting temperatures in the calculated phase diagram of the Ti–Nb–Ag alloy system (Figure 2c,d). Figure 3b shows the equilibrium calculation result for the Ti_53.4_Ag_33.3_Nb_13.3_ alloy. TiAg and Ti_2_Ag intermetallic compounds are seen in a much lower temperature region than the liquidus temperature, and these intermetallic compounds are not formed directly from the liquid at the liquidus temperature. No presence of the ternary intermetallic compounds in the calculated ternary phase diagrams (Figure 2c,d), the predicted ground state diagram (Figure 3a), and the equilibrium calculation results (Figure 3b) indicate that the suppression of LPS via the intermetallic compounds during the cooling of the thermal melt is distant in the Ti_53.4_Ag_33.3_Nb_13.3_ alloy. Based on the abovementioned alloy design and prediction, the solidification microstructure of the ternary Ti_53.4_Ag_33.3_Nb_13.3_ alloy was investigated.

## 4. Results

**Figure 4:**
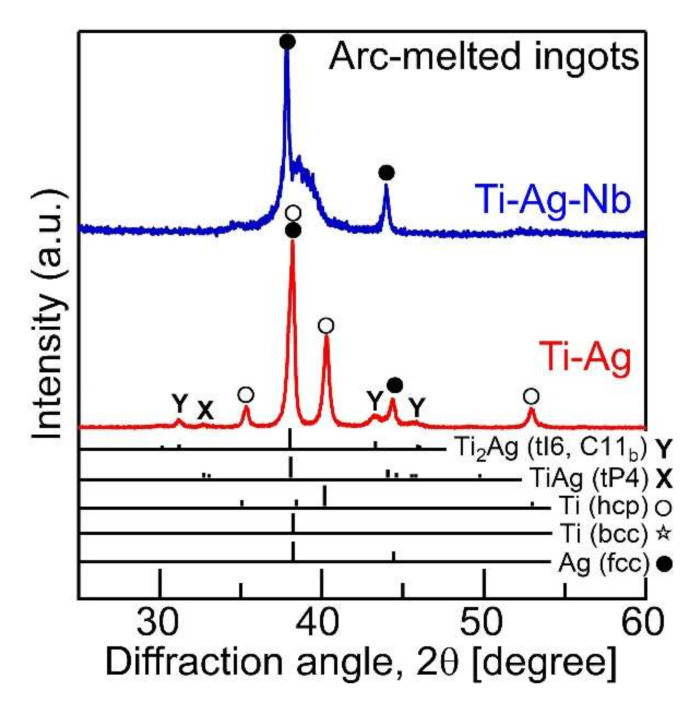
XRD patterns of the arc-melted ingots in the Ti_66.7_Ag_33.3_ (Ti-Ag, red color, lower side) and Ti_53.4_Ag_33.3_Nb_13.3_ (Ti-Nb-Ag, blue color, upper side) alloys.

Figure 4 shows the XRD patterns of the arc-melted ingots in Ti_66.7_Ag_33.3_ (Ti-Ag, red color, lower side) and Ti_53.4_Ag_33.3_Nb_13.3_ (Ti-Nb-Ag, blue color, upper side) alloys, in addition to the calculated intensity of XRD patterns of the Ti with the HCP structure [72], Ti with BCC structure [73], Ag with FCC structure [74], TiAg [67], and Ti_2_Ag [68] intermetallic compounds. The calculated intensity of the XRD patterns was obtained using VESTA [75]. The XRD patterns were obtained from the cross-section of the arc-melted ingots, including the central and copper hearth contacting regions. The formation of the composite of HCP-Ti (○) and FCC-Ag (●) with minor intermetallic compounds of TiAg (X) and Ti_2_Ag (Y) was observed in the arc-melted ingots of the Ti_66.7_Ag_33.3_ alloy [9]. Sharp peaks corresponding to FCC-Ag were observed in the arc-melted ingots of the Ti_53.4_Ag_33.3_Nb_13.3_ alloy, whereas no intermetallic compounds were observed. The broad peak overlapping with the sharp peak of the FCC-Ag (111) was observed only in the Ti_53.4_Ag_33.3_Nb_13.3_ alloy, and the broad peak corresponding to the formation of the martensite phase in the Ti-Nb-rich phase [76,77,78,79,80].

**Figure 5:**
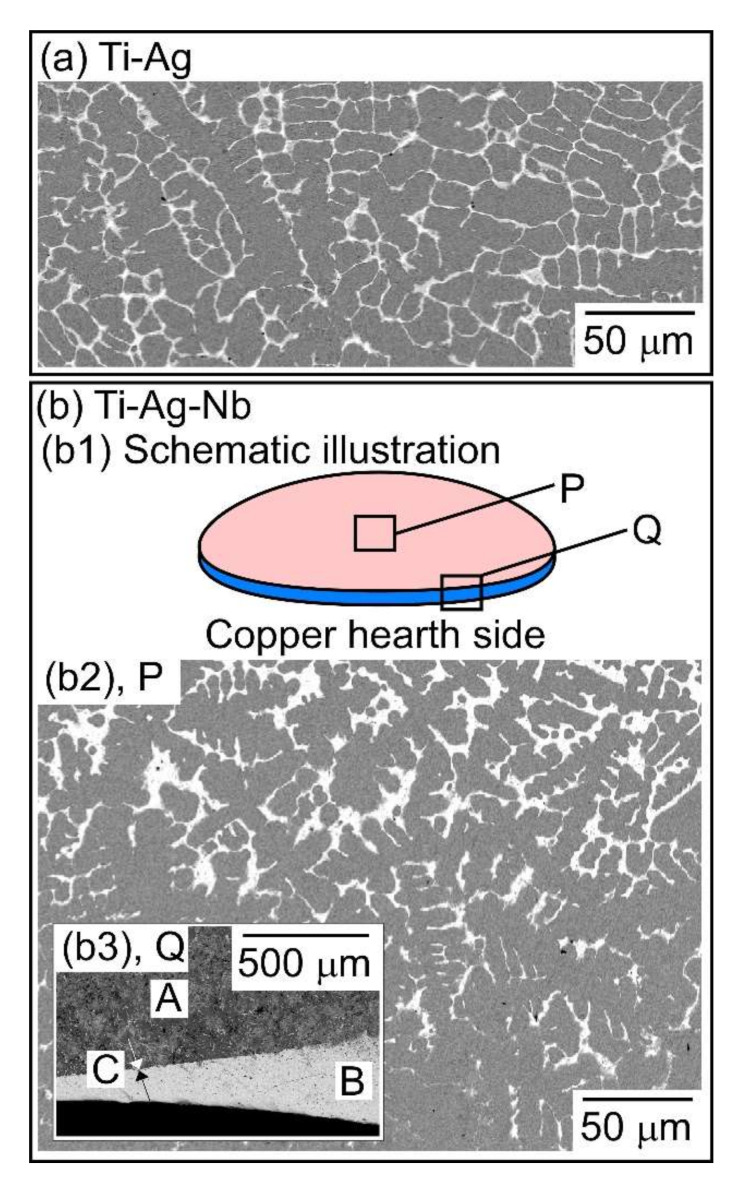
Schematic illustration and SEM-BSE images of the cross-section of arc-melted ingots in the Ti_53.4_Ag_33.3_Nb_13.3_ alloy, together with those of the binary Ti_66.7_Ag_33.3_ alloy as a reference. (a) Ti_66.7_Ag_33.3_ alloy; (**b**) Ti_53.4_Ag_33.3_Nb_13.3_ alloy; (**b1**) Schematic illustration; (**b2**) SEM-BSE image obtained from the central region indicated by index P in Figure 5b1; (**b3**) SEM-BSE image obtained from the bottom region indicated by index Q in Figure 5b1. The lower side corresponds to the copper hearth contacted side.

Figure 5 shows a schematic illustration and SEM-BSE images of the cross-section of arc-melted ingots in the Ti_53.4_Ag_33.3_Nb_13.3_ alloy, together with an SEM-BSE image of arc-melted ingots in the Ti_66.7_Ag_33.3_ alloy as a reference. In the binary Ti_66.7_Ag_33.3_ alloy, a typical equiaxis dendrite structure composed of a gray contrast dendrite and white contrast interdendrite was observed (Figure 5a). A schematic illustration of the macroscopic phase-separated structure in the arc-melted ingots in the Ti_53.4_Ag_33.3_Nb_13.3_ alloy is shown in Figure 5(b1). In the cross section of the arc- melted ingots, the macroscopic phase-separated interface existed at the bottom of the Cu-hearth-contacted side. Figure 5(b2) shows the SEM-BSE image of the central region (the index P in Figure 5(b1) of the ingots. An equiaxis dendrite structure composed of a gray contrast dendrite and white contrast interdendrite, which was similar to the case of the binary Ti_66.7_Ag_33.3_ alloy (Figure 5a), was observed. Figure 5(b3) shows the SEM-BSE image of the bottom (the index Q in Figure 4(b1)) of the ingots. The lower side corresponds to the copper hearth-contacted side. The macroscopic phase-separated interface (the index C in Figure 5(b3)) between the gray contrast (the index A in Figure 5(b3)) and white-contrast regions (index B in Figure 5(b3)) were observed. The micro-solidification structure in the gray contrast region (A in Figure 5(b3)) shows an equiaxis dendrite structure similar to that in Figure 5(b2), whereas the typical dendrite structure was not observed in the macroscopically phase-separated white contrast region (B in Figure 5(b3)).

**Figure 6:**
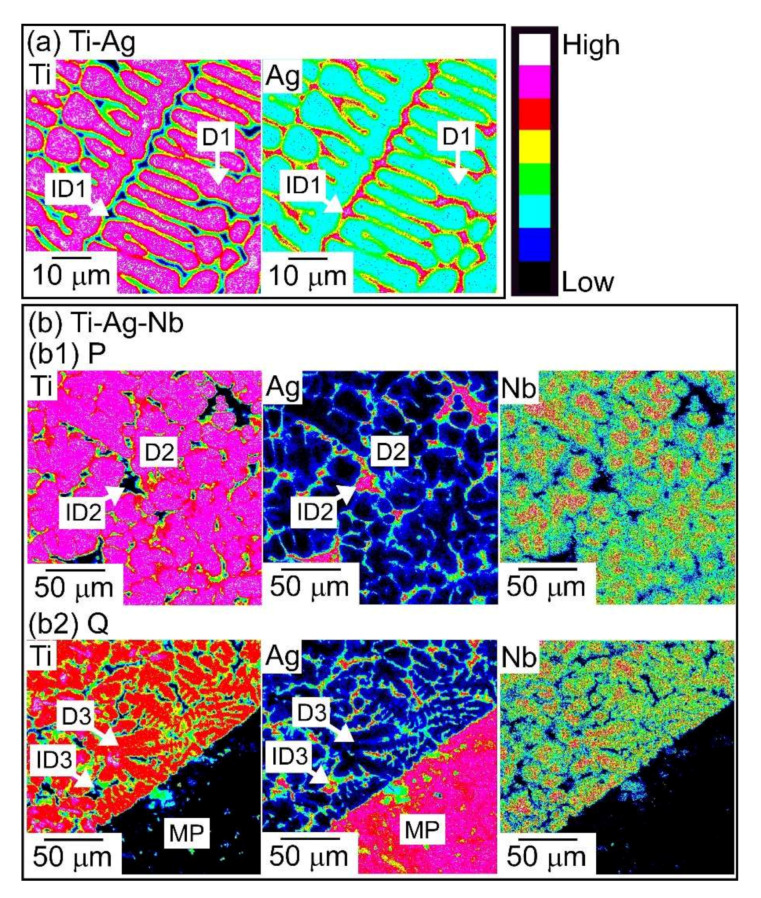
EPMA-WDS elemental mapping of the arc-melted ingots in the Ti_53.4_Ag_33.3_Nb_13.3_ alloy, together with those of the binary Ti_66.7_Ag_33.3_ alloy as a reference. (**a**) Ti_66.7_Ag_33.3_ alloy, (**b**) Ti_53.4_Ag_33.3_Nb_13.3_ alloy; (**b1**) Central region corresponding to index P in Figure 5b1; (**b2**) bottom region corresponding to index Q in Figure 5b1. The indices I, ID, and MP denote the dendrite region, interdendrite region, and macroscopic phase-separated region existed at copper hearth contacted region, respectively.

**Figure 7:**
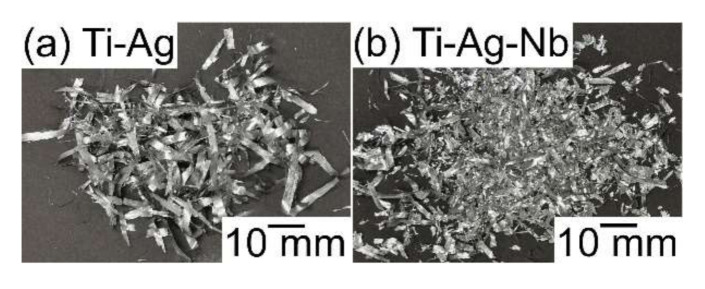
Outer appearances of the rapidly-solidified melt-spun ribbons in the Ti_66.7_Ag_33.3_ (**a**) and Ti_53.4_Ag_33.3_Nb_13.3_ (**b**) alloys.

Figure 6 shows EPMA-WDS elemental mapping of the arc-melted ingots in Ti_53.4_Ag_33.3_Nb_13.3_ alloy, together with EPMA-WDS elemental mapping of arc-melted ingots of the Ti_66.7_Ag_33.3_ alloy as a reference. Table 2 shows the results of the EPMA-WDS analysis of the arc-melted ingots in the Ti_53.4_Ag_33.3_Nb_13.3_ alloy, together with EPMA-WDS analysis of arc-melted ingots in the Ti_66.7_Ag_33.3_ alloy as a reference. Figure 6a shows the elemental mapping of arc-melted ingots in the binary Ti_66.7_Ag_33.3_ alloy. Elemental Ag showed a tendency to be enriched in the interdendrite region, indicated by the index ID1, rather than in the dendrite region, indicated by the index D1. Figure 6(b1) shows the elemental mapping of the central region indicated by the index P in Figure 5(b1). The corresponding SEM-BSE images is shown in Figure 5(b2). In Figure 6(b1), Ti and Nb were enriched at the dendrite phase (D2), while the Ag showed the opposite tendency and was enriched at the interdendrite region (ID2). The dendrite (D2) shows the Ti-rich phase with approximately 80 at %, which contained Nb and Ag in Ti_53.4_Ag_33.3_Nb_13.3_ alloy (Table 2(b2)). The interdendrite (ID2) was an Ag-rich phase with approximately 97 at %, and the solubility of Nb was significantly small (Table 2(b2)). Figure 6(b2) shows the elemental mapping of the macroscopically phase-separated interface embedded at the bottom of arc-melted ingots (index Q in Figure 5(b1)). The gray contrast region indicated by index A in Figure 5(b3) corresponds to the upper left side in the elemental mapping in Figure 6(b2), and the white contrast region indicated by index B in Figure 5(b3) corresponds to the lower right side in the elemental mapping in Figure 6(b2). An equaxis dendrite structure composed of a Ti-Nb-rich dendrite (D3) and Ag-rich interdendrite (ID3) was observed at the upper left side in Figure 6(b2). A significant difference in the chemical composition between the dendrite (D3) in the gray contrast region near the macroscopically phase-separated interfaces shown in Figure 6(b2) and dendrites (D2) at the central region of the ingots shown in Figure 6(b1), was not observed. The similarity in the chemical composition between the interdendrites (ID2 and ID3) in Figure 6(b1,b2) was also observed. The chemical composition of the white contrast region (MP) near the macroscopically phase-separated interfaces in Figure 6(b2), which correspond to region B in Figure 5(b3), is shown in Table 2(b2). The solubility of Ti and Nb in the macroscopically phase-separated region with white contrast in the SEM-BSE image (MP in Figure 6(b2), B in Figure 5(b3)) was significantly small. The dispersion of fine Ag globules via LPS was not observed in the arc-melted ingots in the binary Ti_66.7_Ag_33.3_ and Ti_53.4_Ag_33.3_Nb_13.3_ alloys. Focusing on the differences in the tendency for segregation of Ag in the equiaxis dendrite structure between the binary Ti_66.7_Ag_33.3_ and Ti_53.4_Ag_33.3_Nb_13.3_ alloy, we observed that the Ag/(Ti+Ag) ratio in the interdendtrite region (ID1) of the binary Ti_66.7_Ag_33.3_ alloy (3.7) (Table 2a) was similar to that of the Ag/(Ti+Ag+Nb) ratio in the interdendtrite region (ID2, ID3) of the Ti_53.4_Ag_33.3_Nb_13.3_ alloy (3.0, 3.8) (Table 2(b1,b2)). The particular solidification microstructure of the composite structure with fine Ag globules and a Ti-rich matrix was formed via LPS in the melt-spun ribbons in the binary Ti-Ag alloy [9], was not observed in the arc-melted ingots in ternary Ti-Nb-Ag alloy. The solidification microstructure of the melt-spun ribbons in the Ti_53.4_Ag_33.3_Nb_13.3_ alloy, specifically the morphology of the Ag phase, was investigated. Figure 7 shows the outer appearances of the rapidly melt-spun ribbons in Ti_66.7_Ag_33.3_ and Ti_53.4_Ag_33.3_Nb_13.3_. Flakes, rather than continuous ribbons, were obtained in the melt-spun ribbons in the Ti_53.4_Ag_33.3_Nb_13.3_ alloy (Figure 7b), which was similar to that in the Ti_66.7_Ag_33.3_ alloy (Figure 7a).

**Figure 8:**
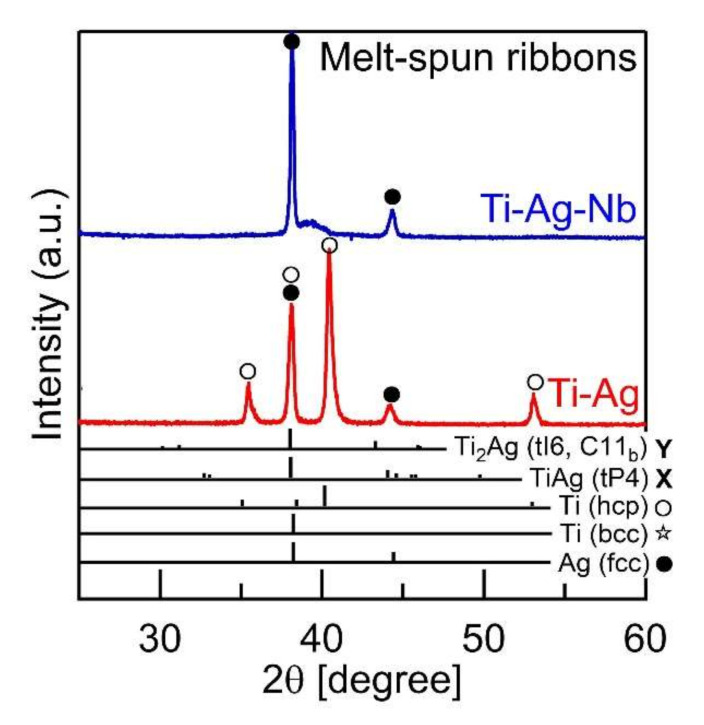
XRD patterns of the rapidly-solidified melt-spun ribbons in the Ti_66.7_Ag_33.3_ (Ti-Ag, red color, lower side) and Ti_53.4_Ag_33.3_Nb_13.3_ (Ti-Nb-Ag, blue color, upper side) alloys.

Figure 8 shows the XRD patterns of the melt-spun ribbons in the Ti_66.7_Ag_33.3_ (Ti-Ag, red color, lower side) and Ti_53.4_Ag_33.3_Nb_13.3_ (Ti-Ag-Nb, blue color, upper side) alloys. In the Ti_66.7_Ag_33.3_ alloy, sharp peaks corresponding to the Ti–Ag intermetallic compounds were not observed, indicating that rapid solidification was effective in suppressing intermetallic compound formation. The composite of HCP-Ti and FCC-Ag formed in the melt-spun ribbons in the Ti_66.7_Ag_33.3_ alloy. Sharp peaks corresponding to FCC-Ag and broad peaks, whose position overlapped with that of Ag (111), were observed in the melt-spun ribbons of the Ti_53.4_Ag_33.3_Nb_13.3_ alloy. The origin of the broad peaks in the melt-spun ribbons was not clarified in the present study, and can be considered to be related to the martensite phase formation in the Ti-Nb alloys [76,77,78,79,80]. Further identification of the broad peak in the XRD patterns with electron microscopy can be conducted in the future. The sharp peaks corresponding to the intermetallic compounds and HCP-Ti were not observed in the Ti_53.4_Ag_33.3_Nb_13.3_ alloy.

**Figure 9:**
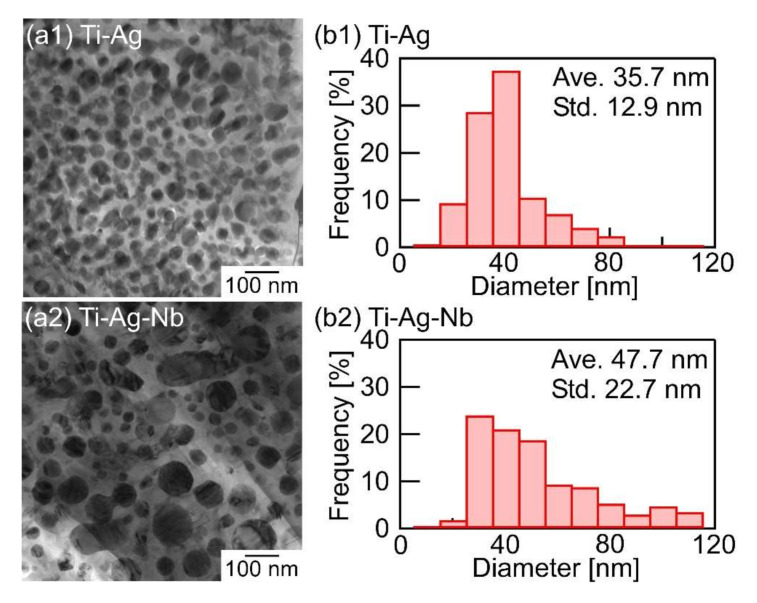
TEM-BF images and the size distribution of the globules in the rapidly-solidified melt-spun ribbons in Ti_53.4_Ag_33.3_Nb_13.3_, together with those of the binary Ti_66.7_Ag_33.3_ alloy as a reference. (**a**) TEM-BF images, (**b**) the size distribution of the globules. (**a1**,**b1**) Ti_66.7_Ag_33.3_ alloy, (**a2**,**b2**) Ti_53.4_Ag_33.3_Nb_13.3_ alloy. The denotations of Ave. and Std. refer to the average size and the standard deviation of the size of globules, respectively.

Figure 9 shows the TEM-bright field (BF) image and size distribution analysis of the globules of melt-spun ribbons in the Ti_53.4_Ag_33.3_Nb_13.3_ alloy and in the binary Ti_66.7_Ag_33.3_ alloy. The globules and connected globules with crystalline contrasts were embedded in the gray contrast matrix phase of the Ti_53.4_Ag_33.3_Nb_13.3_ alloy (Figure 9(a2)), and the morphology was similar to that of the Ti_66.7_Ag_33.3_ alloy (Figure 9(a1)). The size distribution of globules was analyzed using the inter-linear method, and the results are shown in Figure 9(b1,b2). In Figure 9b, the denotations of Ave. and Std. refer to the average size and standard deviation of the size of globules, respectively. The average size of the globules in the Ti_53.4_Ag_33.3_Nb_13.3_ alloy (47.7 nm) was larger than that in the Ti_66.7_Ag_33.3_ alloy (35.7 nm). The size distribution of the globules in the Ti_53.4_Ag_33.3_Nb_13.3_ alloy (Figure 9(b2)) was wider than that in the Ti_66.7_Ag_33.3_ alloy (Figure 9(b1)). Globules with diameters greater than 100 nm were observed in the Ti_53.4_Ag_33.3_Nb_13.3_ alloy, while such large globules were not observed in the Ti_66.7_Ag_33.3_ alloy. The addition of Nb to the Ti-Ag alloy affects the size distribution of globules in the rapidly solidified melt- spun ribbons.

**Figure 10:**
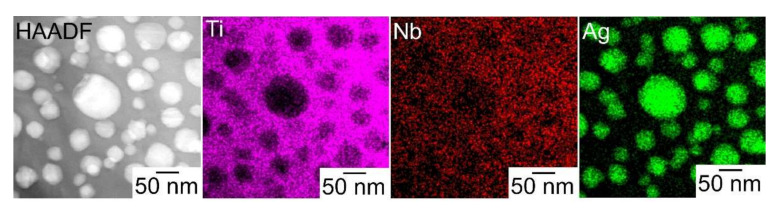
STEM-HAADF image and STEM-EDS elemental mapping of the rapidly-solidified melt-spun ribbons in the Ti_53.4_Ag_33.3_Nb_13.3_ alloy.

Figure 10 shows the STEM high-angle annular dark-field scanning (HAADF) image and STEM energy dispersive X-ray spectroscopy (EDS) elemental mapping of the melt-spun ribbons in the Ti_53.4_Ag_33.3_Nb_13.3_ alloy [10]. In the STEM-HAADF image, the globules with white contrasts were embedded in the gray contrast matrix. In the STEM EDS mapping, Ag (atomic number (Z) = 47) was enriched in the globules, while Ti (Z = 22) and Nb (Z = 41) were enriched in the matrix. The chemical composition of the globules and matrix evaluated using EDS are shown in Table 3. The Ag concentration of the Ag-rich globules was over 92 at.%, and the solubility of Nb was significantly small. Based on the XRD analysis (Figure 8), TEM (Figure 9), STEM (Figure 10), STEM-EDS (Table 3) in the melt-spun ribbons, and the formation of fine Ag globule-dispersed Ti-Nb alloy via rapid solidification were confirmed in the alloy. The microstructure consists of fine FCC-Ag globules and a Ti-based matrix achieved in Ti-Nb-Ag alloys.

## 5. Discussion

A macroscopically phase-separated structure was observed in the arc-melted ingots of the Ti_53.4_Ag_33.3_Nb_13.3_ alloy using SEM observation (Figure 5) and EPMA analysis (Figure 6 and Table 2). The formation of the macroscopically phase-separated Ag phase at the bottom of the ingots was not observed in the Ti_66.7_Ag_33.3_ alloy [9]. The difference in the solidification microstructures in arc-melted ingots between the Ti_66.7_Ag_33.3_ and Ti_53.4_Ag_33.3_Nb_13.3_ alloys indicated the accuracy of the thermodynamic calculation prediction that the addition of Nb enhanced the LPS in the Ti-Ag alloy system (Figure 2).

**Figure 11:**
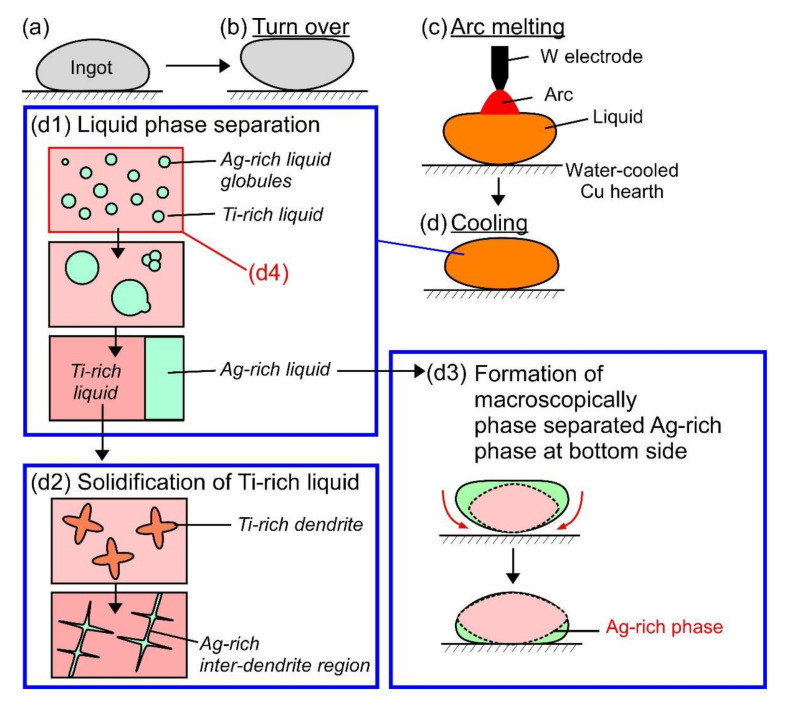
Schematic illustration of the mechanism of the macroscopically phase-separated structure in the arc-melted ingots in the Ti_53.4_Ag_33.3_Nb_13.3_ alloy and composite of fine Ag globules and Ti-Nb matrix in the melt-spun ribbons. (**a**) before arc-melting, (**b**) turning over of ingot before arc melting, (**c**) arc melting, (**d**) cooling of the thermal melt, (**d1**) liquid phase separation and aggregation of minor Ag rich liquid globules, (**d2**) solidification of Ti-rich liquid, (**d3**) formation of macroscopically phase-separated Ag-rich phase at the bottom, and (**d4**) the state after the liquid phase separation without starting the aggregation of minor Ag rich liquid globules.

Figure 11 shows a schematic illustration of the mechanism of the macroscopically phase-separated structure in the arc-melted ingots in the Ti_53.4_Ag_33.3_Nb_13.3_ alloy. Figure 11a–d shows the schematic illustration of the arc-melting sequences: (a) before arc-melting, (b) turning over of the ingot before arc melting, (c) arc melting, and (d) cooling of the thermal melt after arc melting. Figure 11(d1) shows a schematic illustration of the LPS and aggregation of the minor Ag-rich liquid globules process. LPS leads to the formation of a composite of the main Ti-rich liquid matrix and minor Ag-rich liquid globules (Figure 11(d4)). The aggregation of the Ag-rich liquid globules progressed during the cooling of the thermal melt, which resulted in the macroscopically phase- liquid formed via LPS. The Ti-Nb-rich dendrite formed through the crystallization of the Ti-rich and rejection of Ag from the dendrite to the residual liquid. This resulted in the formation of the Ag-rich separated Ti-rich and Ag-rich liquids when there was sufficient time for the aggregation of fine Ag-rich liquid globules. Figure 11(d2) shows a schematic illustration of the solidification of the Ti-rich residual liquid in the interdendrite region. The segregation of Ag leads to the formation of the Ag-rich interdendrite phase, resulting in the formation of an equiaxis dendrite structure composed of a Ti-Nb-rich dendrite and Ag-rich interdendritic phases. The formation of the macroscopically phase-separated structure with the Ag-rich phase in contact with the copper hearth at the bottom side was characterized as the arc-melting process. Figure 11(d3) shows a schematic illustration of the formation of the macroscopically phase-separated Ag-rich phase at the bottom of the ingots. The melting temperature of the separated Ag-rich liquid was lower than that of the Ti-Nb-rich liquid, as shown in the pseudo-binary phase diagram in Figure 2f. The Ag-rich liquid flowed to the bottom of the arc-melted ingots, resulting in the formation of the macroscopically phase-separated structure at the bottom of the ingots. The thermodynamic calculation (Figure 3b) implies the formation of Ti_2_Ag and TiAg intermetallic compounds during the cooling of the ingot right after solidification, while the formation of intermetallic compounds as the main constituent phases was not detected in the arc-melted ingots. The difference in the constituent phases between the thermodynamic calculations and experimental observations can be explained by LPS through the formation of Ti-Nb- and Ag-rich liquids, segregation of Ag during the solidification of the Ti-Nb-rich liquid, and martensitic transformation during the cooling of Ti-Nb-rich dendrites. The FCC-Ag phase was formed via LPS and segregation during solidification, resulting in the formation of Ti-Nb-rich dendrites with low Ag concentration. Ti_2_Ag and TiAg intermetallic compounds were not formed in Ti-Nb-rich dendrites due to the lack of Ag.

The fine Ag-dispersed Ti-Nb alloy was obtained in the melt-spun ribbons in the Ti_53.4_Ag_33.3_Nb_13.3_ alloys via LPS and rapid solidification. The rapid solidification during the melt-spinning process was considered to be effective for the formation of fine Ag globules embedded in the Ti–Nb matrix for the following reasons: (1) the aggregation of Ag globules formed via LPS was suppressed by the rapid cooling of the thermal melt. In other words, the morphology of the Ag globules shown in Figure 11(d4) was frozen during the rapid cooling of the thermal melt, resulting in the fine Ag globules dispersed in the Ti-Nb matrix being obtained only in the melt-spun ribbons. (2) The LPS with supercooling was achieved using the melt-spinning process. Supercooling was effective in decreasing the size of the Ag globules. This paper demonstrates that ternary Ti-based alloys with fine Ag phases can be fabricated by a casting process with LPS as well as powder metallurgy [41]. The results for the fabrication of the composite of the fine Ag globule-dispersed Ti-Nb alloy are important for the further development of Ti-Ag based alloys, which are widely investigated as dental materials [24,25,26,27,28,29,30,31,32,33,34] and/or metallic antibacterial materials [35,36,37,38,39,40]. The prediction of LPS using mixing enthalpy (Figure 1), thermodynamic calculation (Figure 2, Figure 3b and Table 1), and predicted ground state diagram (Figure 3a), and experimental observation of the difference in the morphology of Ag between the arc-melted ingots (Figure 4, Figure 5 and Figure 6, and Table 2), and rapidly solidified melt-spun ribbons (Figure 7, Figure 8, Figure 9 and Figure 10, and Table 3) offer a unique opportunity for designing composites with Ag globules dispersed in Ti-based metal matrix in Ti-Ag-based alloys. The dispersion of fine globules was reported to be effective for the strengthening of Ti-rare earth-based alloys [1,2,3,4,5,6]. The fine Ag dispersion may be effective for increasing the mechanical strength. This study shows the possibility of a new structural control method for increasing the mechanical strength of Ti-based dental materials and/or metallic antibacterial materials.

## 6. Conclusions

Fine Ag globule-dispersed Ti–Ag-based Ti–Ag–Nb immiscible alloys with liquid phase separation (LPS) were developed. The first finding of LPS in the Ti–Ag–Nb alloy offers a unique opportunity in designing composites with fine globules dispersed in a Ti-based metal matrix in Ti-based alloys. The obtained results can be summarized as follows:

(1) Ternary Ti–Ag-based alloys with LPS was discussed based on the mixing enthalpy of the constituent elements, thermodynamic calculation, and predicted phase diagrams constructed by the Materials Project.
(2) LPS was experimentally found in the ternary Ti–Ag-Nb alloy of Ti_53.4_Ag_33.3_Nb_13.3_.
(3) A macroscopically phase-separated structure was observed in the arc-melted ingots, where the cooling rate of arc-melting was approximately 2000 K/s. The macroscopically phase-separated structure Ag-rich phase existed in the copper hearth contacted region in the arc-melted ingots. In the central region of the ingots, an equiaxis dendrite structure composed of a Ti-rich dendrite and Ag-rich interdendrite was observed.
(4) A composite of fine Ag globules and Ti–Nb-based alloy matrix was obtained in rapidly solidified melt-spun ribbons in the Ti_53.4_Ag_33.3_Nb_13.3_ alloy. Super-cooling of the thermal melt leads to LPS and the suppression of the aggregation of Ag-rich liquid globules, resulting in the formation of a particular microstructure comprising 100 nm order Ag globules and Ti alloy matrix.

## Figures and Tables

**Table 1:** Thermodynamic calculation results of the composition of the separated liquids in the Ti_53.4_Ag_33.3_Nb_13.3_ alloy. (**a**) Ti-rich liquid; (**b**) Ag-rich liquid.

Phases	Temperature (K)	Ti	Ag	Nb
(a) Ti-rich liquid	1900	57.4	27.1	15.4
	1800	61.4	21.6	16.9
	1700	64.5	17.7	17.8
	1400	71.6	9.4	19.0
(b) Ag-rich liquid	1900	37.7	57.0	5.3
	1800	30.6	66.3	3.2
	1700	24.4	73.7	1.9
	1400	11.2	88.4	0.4

**Table 2:** EPMA-WDS analysis of arc-melted ingots of the Ti_53.4_Ag_33.3_Nb_13.3_ alloy, together with the data of the binary Ti_66.7_Ag_33.3_ alloy as a reference. The corresponding positions of the measured area are shown in Figure 5. (**a**) Ti_66.7_Ag_33.3_ alloy, (b) Ti_53.4_Ag_33.3_Nb_13.3_ alloy, (**b1**) Central region indicated by the index P in Figure 5(b1), and (**b2**) Bottom region indicated by the index Q in Figure 5(b1).

Regions	Regions	Ti	Ag	Nb
(a) Ti-Ag	D1 (dendrite)	82.0	18.0	-
	ID1 (interdenbdrite)	3.7	96.3	-
(b1) Ti-Ag-Nb, region P (central part)	D2 (dendrite)	78.0	10.9	11.1
	ID2 (interdenbdrite)	3.0	96.7	0.3
(b2) Ti-Ag-Nb, region Q (bottom part)	D3 (dendrite)	78.7	10.7	10.6
	ID3 (interdenbdrite)	3.8	95.9	0.3
	MP	0.5	99.4	0.

**Table 3:** STEM-EDS analysis results of the rapidly-solidified melt-spun ribbons in the Ti_53.4_Ag_33.3_Nb_13.3_ alloy.

Regions	Ti	Ag	Nb
Matrix	75.4	9.6	15.0
Globules	6.9	92.4	0.8

## References

[1] Sastry S.M.L., Peng T.C., Beckerman L.P. (1984). Structure and properties of rapidly solidified dispersion strengthened titanium alloys: Part II. tensile and creep properties. Metall. Trans. A.

[2] Whang S.H. (1986). Review—Rapidly solidified titanium alloys for high-temperature applications. J. Mater. Sci..

[3] Court S.A., Sears J.W., Loretto M.H., Fraser H.L. (1988). The Effect of Liquid Phase Separation on the Microstructure of Rapidly Solidified Titanium-Rare Earth Alloys. Mater. Sci. Eng..

[4] Suryanarayana C., Froes F.H., Rowe R.G. (1991). Rapid solidification processing of titanium alloys. Inter. Mater. Rev..

[5] Gigliotti M.F.X., Woodfield A.P. (1993). The Roles of Rare Earth Dispersoids and Process Route on the Low Cycle Fatigue Behavior of a Rapidly Solidified Powder Metallurgy Titanium Alloy. Metall. Mater. Trans. A.

[6] Kral M.V., Hofmeister W.H., Witting J.E. (1997). Interphase Boundary Precipitation in a Ti-1.7 At. Pct Er Alloy. Metall. Mater. Trans. A.

[7] Ward-Close C.M., Partridge P.G. (1991). The production of titanium-magnesium alloys by vapour quenching. Mater. Lett..

[8] Ward-Close C.M., Lu G., Partridge P.G. (1994). Microstructure of vapour-quenched Ti-Mg alloys. Mater. Sci. Eng. A.

[9] Nagase T., Matsumoto M., Fujii Y. (2018). Microstructure of Ti-Ag immiscible alloys with metastable liquid phase separation. J. Alloys Compd..

[10] Nagase T., Matsumoto M., Fujii Y. (2017). Microstructure of Ti-Nb-Ag immiscible alloys with liquid phase separation. Microscopy.

[11] Massalski T.B., Okamoto H., Subramanian P.R. (1990). Binary Alloy Phase Diagrams, Ti-Y.

[12] Massalski T.B., Okamoto H., Subramanian P.R. (1990). Binary Alloy Phase Diagrams, La-Ti.

[13] Massalski T.B., Okamoto H., Subramanian P.R. (1990). Binary Alloy Phase Diagrams, Ce-Ti.

[14] Massalski T.B., Okamoto H., Subramanian P.R. (1990). Binary Alloy Phase Diagrams, Pr-Ti.

[15] Massalski T.B., Okamoto H., Subramanian P.R. (1990). Binary Alloy Phase Diagrams, Nd-Ti.

[16] Massalski T.B., Okamoto H., Subramanian P.R. (1990). Binary Alloy Phase Diagrams, Gd-Ti.

[17] Massalski T.B., Okamoto H., Subramanian P.R. (1990). Binary Alloy Phase Diagrams, Dy-Ti.

[18] Massalski T.B., Okamoto H., Subramanian P.R. (1990). Binary Alloy Phase Diagrams, Ti-Yb.

[19] Massalski T.B., Okamoto H., Subramanian P.R. (1990). Binary Alloy Phase Diagrams, Mg-Ti.

[20] Takeuchi A., Inoue A. (2000). Calculations of Mixing Enthalpy and Mismatch Entropy for Ternary Amorphous Alloys. Mater. Trans..

[21] Takeuchi A., Inoue A. (2005). Classification of Bulk Metallic Glasses by Atomic Size Difference, Heat of Mixing and Period of Constituent Elements and Its Application to Characterization of the Main Alloying Element. Mater. Trans..

[22] Li M., Li C., Wang F., Zhang W. (2005). Experimental study and thermodynamic assessment of the Ag-Ti system. Calphad.

[23] Massalski T.B., Okamoto H., Subramanian P.R. (1990). Ag-Ti. Binary Alloy Phase Diagrams.

[24] Takada Y., Nakajima H., Okuno O., Okabe T. (2001). Microstructure and Corrosion Behavior of Binary Titanium Alloys with Beta-stabilizing Elements. Dent. Mater. J..

[25] Takahashi M., Kikuchi M., Takada Y., Okuno O. (2002). Mechanical Properties and Microstructures of Dental Cast Ti-Ag and Ti-Cu Alloys. Dent. Mater. J..

[26] Kikuchi M., Takahashi M., Okabe T., Okuno O. (2003). Grindability of Dental Cast Ti-Ag and Ti-Cu Alloys. Dent. Mater. J..

[27] Oh K.-T., Shim H.-M., Kim K.-N. (2005). Properties of titanium-silver alloys for dental application. J. Biomed. Mater. Res. Part B Appl. Biomater..

[28] Kikuchi M., Takahashi M., Okuno O. (2008). Machinability of Experimental Ti-Ag Alloys. Dent. Mater. J..

[29] Zhang B.B., Zheng Y.F., Liu Y. (2009). Effect of Ag on the corrosion behavior of Ti-Ag alloys in artificial saliva solutions. Dent. Mater..

[30] Takahashi M., Kikuchi M., Takada Y., Okuno O. (2010). Corrosion Resistance of Dental Ti-Ag Alloys in NaCl Solution. Mater. Trans..

[31] Zhang B.B., Qiu K.J., Wang B.L., Li L., Zheng Y.F. (2012). Surface Characterization and Cell Response of Binary Ti-Ag Alloys with CP Ti as Material Control. J. Mater. Sci. Technol..

[32] Takahashi M., Kikuchi M., Takada Y. (2015). Mechanical properties of dental Ti-Ag alloys with 22.5, 25, 27.5, and 30 mass% Ag. Dent. Mater. J..

[33] Han M.-K., Hwang M.-J., Won D.-H., Kim Y.-S., Song H.-J., Park Y.-J. (2014). Massive Transformation in Titanium-Silver Alloys and Its Effect on Their Mechanical Properties and Corrosion Behavior. Materials.

[34] Nakajo K., Takahashi M., Kikuchi M., Takada Y., Okuno O., Sasaki K., Takahashi N. (2014). Inhibitory effect of Ti-Ag alloy on artificial biofilm formation. Dent. Mater. J..

[35] Liu X., Tian A., You J., Zhang H., Wu L., Bai X., Lei Z., Shi X., Xue X., Wang H. (2016). Antibacterial abilities and biocompatibilities of Ti-Ag alloys with nanotubular coatings. Int. J. Nanomed..

[36] Liu X., Chen S., Tsoi J.K.H., Matinlinna J.P. (2017). Binary titanium alloys as dental implant materials-a review. Regen. Biomater..

[37] Lei Z., Zhang H., Zhang E., You J., Ma X., Bai X. (2018). Antibacterial activities and biocompatibilities of Ti-Ag alloys prepared by spark plasma sintering and acid etching. Mater. Sci. Eng. C.

[38] Al-Rawy W.A., Al-Hassani E.S. (2019). Effect of Ag Addition on Cp-Ti Dental Implant.

[39] Shi A., Zhu C., Fu S., Wang R., Qin G., Chen D., Zhang E. (2020). What controls the antibacterial activity of Ti-Ag alloy, Ag ion or Ti_2_Ag particles?. Mater. Sci. Eng. C.

[40] Lei Z., Zhang H., Zhang E., You J., Ma X., Bai X. (2020). Antibacterial activities and cell responses of Ti-Ag alloys with a hybrid micro- to nanostructured surface. J. Biomater. Appl..

[41] Wen M., Wen C., Hodgson P., Li Y. (2014). Fabrication of Ti-Nb-Ag alloy via powder metallurgy for biomedical applications. Mater. Des..

[42] Sdobnyakov N.Y., Myasnichenko V.S., San C.-H., Chiu Y.-T., Ershov P.M., Ivanov V.A., Komarov P.V. (2019). Simulation of phase transformations in titanium nanoalloy at different cooling rates. Mater. Chem. Phys..

[43] Myasnichenko V.S., Sdobnyakov N.Y., Ershov P.M., Sokolov D.N., Kolosov A.Y., Davydenkova E.M. (2020). Simulation of Crystalline Phase Formation in Titanium-Based Bimetallic Clusters. J. Nano Res..

[44] Aguilar C., Martinez C., Tello K., Palma S., Delonca A., Martin F.S., Alfonso I. (2020). Thermodynamic Analysis of the Formation of FCC and BCC Solid Solutions of Ti-Based Ternary Alloys by Mechanical Alloying. Metals.

[45] Nagase T., Mizuuchi K., Nakano T. (2019). Solidification Microstructures of the Ingots Obtained by Arc Melting and Cold Crucible Levitation Melting in TiNbTaZr Medium-Entropy Alloy and TiNbTaZrX (X = V, Mo, W) High-Entropy Alloys. Entropy.

[46] Nagase T., Takemura M., Matsumuro M., Maruyama T. (2018). Solidification Microstructure of AlCoCrFeNi2.1 Eutectic High Entropy Alloy Ingots. Mater. Trans..

[47] Nagase T., Kakeshita T., Matsumura K., Nakazawa K., Furuya S., Ozoe N., Yoshino K. (2019). Development of Fe-Co-Cr-Mn-Ni-C high entropy cast iron (HE cast iron) available for casting in air atmosphere. Mater. Des..

[48] Cahn R.W. (1983). Physical Metallurgy.

[49] Miyake H., Furusawa A., Ariyasu T., Okada A. (1994). Optical Measurement of Cooling Rate During Splat Cooling Process. J. Jpn. Foundar. Soc..

[50] FactSage Home Page. http://www.factsage.com/.

[51] SGTE—SGTE 2017 Alloy Phase Diagrams (1176) Home Page. http://www.crct.polymtl.ca/fact/documentation/SGTE2017/SGTE2017_Figs.htm.

[52] Nagase T., Suzuki M., Tanaka T. (2015). Formation of amorphous phase with crystalline globules in Fe-Cu-Nb-B immiscible alloys. J. Alloys Compd..

[53] Nagase T., Suzuki M., Tanaka T. (2015). Formation of amorphous phase with crystalline globules in Fe-Cu-Si-B and Fe-Cu-Zr-B immiscible alloys. Intermetallics.

[54] Nagase T., Suzuki M., Tanaka T. (2015). Amorphous phase formation in Fe-Ag-based immiscible alloys. J. Alloys Compd..

[55] Nagase T., Takemura M., Matsumuro M., Matsumoto M., Fujii Y. (2017). Design and microstructure analysis of globules in Al-Co-La-Pb immiscible alloys with an amorphous phase. Mater. Des..

[56] Nagase T., Todai M., Nakano T. (2020). Development of Co-Cr-Mo-Fe-Mn-W and Co-Cr-Mo-Fe-Mn-W-Ag High-Entropy Alloys Based on Co-Cr-Mo alloys. Mater. Trans..

[57] Nagase T., Todai M., Nakano T. (2020). Liquid Phase Separation in Ag-Co-Cr-Fe-Mn-Ni, Co-Cr-Cu-Fe-Mn-Ni and Co-Cr-Cu-Fe-Mn-Ni-B High Entropy Alloys for Biomedical Application. Crystals.

[58] Steinemann S.G. (1980). Evaluation of Biomaterials.

[59] Kawahara H. (1992). Cytotoxicity of Implantable Metals and Alloys. Bull. Jpn. Inst. Metals..

[60] Okazaki Y. (1998). Cytocompatibility of Various Metals and Development of New Titanium Alloy for Medical Implant. Mater. Jpn..

[61] Narushima T. (2005). Titanium and its alloys as biomaterials. J. Jpn. Inst. Light Metals..

[62] Hopkins R.H., Stewart A.M., Daniel M.R. (1978). Phase relations and a15-phase diffusion layer formation in the system Ag-Nb-Ga. Metall. Mater. Trans..

[63] Subramanian P.R., Simmons J.P. (1991). Phase equilibria in the vicinity of the DO_22_ Al_3_Nb composition in the Al-Nb-W, Al-Nb-Co, Al-Nb-Pt, and Al-Nb-Ag systems. Scripta Metall. Mater..

[64] Jain A., Ong S.P., Hautier G., Chen W., Richards W.D., Dacek S., Cholia S., Gunter D., Skinner D., Ceder G. (2013). The Materials Project: A materials genome approach to accelerating materials innovation. APL Mater..

[65] Materials Projects Home Page. https://materialsproject.org/.

[66] Nagase T., Todai M., Nakano T. (2020). Development of Ti-Zr-Hf-Y-La high-entropy alloys with dual hexagonal-close-packed structure. Scr. Mater..

[67] (2013). Materials Projects, TiAg, ID: Mp-1017985.

[68] (2013). Materials Projects, Ti2Ag, ID: Mp-979115.

[69] Antonova N.V., Firstov S.A., Miracle D.B. (2003). Investigation of phase equilibria in the Ti-Al-Si-Nb system at low Nb contents. Acta Mater..

[70] Okamoto H. (2002). Nb-Ti (Niobium-Titanium). J. Ph. Equilib..

[71] Massalski T.B., Okamoto H., Subramanian P.R. (1990). Binary Alloy Phase Diagrams, Nb-Ti.

[72] (2013). Materials Projects, Ti (HCP), ID: Mp-46.

[73] (2013). Materials Projects, Ti (BCC), ID: Mp-73.

[74] (2013). Materials Projects, Ag, ID: Mp-124.

[75] Momma K., Izumi F. (2008). VESTA: A three-dimensional visualization system for electronic and structural analysis. J. Appl. Crystallogr..

[76] Lee C.M., Ju C.P., Lin J.H.C. (2002). Structure-property relationship of cast Ti–Nb alloys. J. Oral Rehabilitation..

[77] Mantani Y., Tajima M. (2006). Phase transformation of quenched a’’ martensite by aging in Ti–Nb alloys. Mater. Sci. Eng. A..

[78] Banumathy S., Mandal R.K., Singh A.K. (2009). Structure of orthorhombic martensitic phase in binary Ti–Nb alloys. J. Appl. Phys..

[79] Bonisch M., Calin M., Waitz T., Panigrahi A., Zehetbauer M., Gebert A., Skrotzki W., Eckert J. (2013). Thermal stability and phase transformations of martensitic Ti–Nb alloys. Sci. Technol. Adv. Mater..

[80] Mantani Y., Takemoto Y. (2015). Change in Crystal Structure and Material Properties with Deformation of Quenched Martensite in Ti-Nb Alloys. J. Jpn. Inst. Met. Mater..

